# Vaccine Hesitancy during the Coronavirus Pandemic in South Tyrol, Italy: Linguistic Correlates in a Representative Cross-Sectional Survey

**DOI:** 10.3390/vaccines10101584

**Published:** 2022-09-21

**Authors:** Verena Barbieri, Christian J. Wiedermann, Stefano Lombardo, Dietmar Ausserhofer, Barbara Plagg, Giuliano Piccoliori, Timon Gärtner, Wolfgang Wiedermann, Adolf Engl

**Affiliations:** 1 Institute of General Practice and Public Health, Claudiana College of Health Professions, 39100 Bolzano, Italy; 2 Department of Public Health, Medical Decision Making and Health Technology Assessment, University of Health Sciences, Medical Informatics and Technology, 6060 Hall, Austria; 3 Provincial Institute for Statistics of the Autonomous Province of Bolzano—South Tyrol (ASTAT), 39100 Bolzano, Italy; 4 Faculty of Education, Free University of Bolzano, 39100 Bolzano, Italy; 5 Department of Educational, School and Counseling Psychology, College of Education and Human Development, Missouri Prevention Science Institute, University of Missouri, Columbia, MO 65211, USA

**Keywords:** COVID-19, vaccination, hesitancy, linguistic minorities

## Abstract

Background: German is a minority language in Italy and is spoken by the majority of the inhabitants of the Autonomous Province of Bolzano, South Tyrol. Linguistic group membership in South Tyrol is an established determinant of health information-seeking behavior. Because the COVID-19 incidence and vaccination coverage in the second year of the pandemic in Italy was the worst in South Tyrol, we investigated whether linguistic group membership is related to COVID-19 vaccine hesitancy. Methods: A cross-sectional survey was conducted on a probability-based sample of 1425 citizens from South Tyrol in March 2021. The questionnaire collected information on socio-demographics, including linguistic group membership, comorbidities, COVID-19-related experiences, conspiracy thinking, well-being, altruism, and likelihood of accepting the national vaccination plan. Multiple logistic regression analyses were performed to identify the significant predictors of vaccine hesitancy. Results: Overall, 15.6 percent of the sample reported vaccine hesitancy, which was significantly higher among German speakers than among other linguistic groups. Increased hesitancy was mostly observed in young age, the absence of chronic disease, rural residence, a worsened economic situation, mistrust in institutions, and conspiracy thinking. In the multiple logistic regression analyses, linguistic group membership was not an independent predictor of vaccine hesitancy. Conclusion: Although German is a minority language in Italy and COVID-19 vaccine hesitancy was higher in the German native language group than in the Italian, linguistic group membership was not an independent predictor of hesitancy in the autonomous province. Known predictors of vaccine hesitancy are distributed unevenly across language groups. Whether language group-specific intervention strategies to promote vaccine hesitancy are useful requires further study.

## 1. Introduction

SARS-CoV-2 (severe acute respiratory syndrome coronavirus 2) has led to a global pandemic and has manifested as coronavirus disease 2019 (COVID-19). Safe and effective vaccination for COVID-19 is seen as a long-term solution to the pandemic; however, vaccination rates vary substantially between countries. Regulatory quality is among the most important indicators predicting COVID-19 vaccination status in a country [1]; however, vaccine uptake is impacted by many additional factors, including personal beliefs; cultural, religious, or moral values; vaccination access; anecdotes; and previous vaccination experiences [2]. Data on the effects of race and ethnicity on infection rates and disease severity of COVID-19 exposed a significant impact of health disparities at both the global and national levels, including among minorities in Western countries [3].

According to Our World in Data [4], the first-dose vaccination rates in five central European countries (Slovenia, Austria, Switzerland, France, and Italy) varied considerably (between 60 and 82 per cent) despite the fact that these countries are neighbors with similarly good governance indicators (Supplementary Material Table S1). Therefore, it is critical to better understand what determines vaccine uptake, including vaccination intention, and the factors that could determine COVID-19 vaccine hesitancy and resistance. A cross-national representative survey of over 7000 participants in seven European countries (Denmark, France, Germany, Italy, Portugal, the Netherlands, and the UK) reported that, as with vaccine uptake, there was substantial variation in vaccine hesitancy between countries, ranging from 12 to 28 per cent [5].

Vaccine hesitancy may not be uniformly distributed in large, single countries. In Black communities in the United Kingdom [6] and the United States [7] for instance, a historical mistrust in the government and medical authorities has been a deterrent. On 17 January 2022, in Italy, which was among the leading countries in terms of vaccine uptake in Europe among 21 regions, the first-dose vaccination rates ranged between 74 and 82 per cent. In Italy, the vaccination rate was the lowest in South Tyrol (Supplementary Material Table S2), to which vaccine hesitancy and resistance could be contributing factors. South Tyrol (total population of around 525,000) is located in the alpine part of the northwestern region of Trentino–Alto Adige in Italy and is located at the border of Austria, with approximately 70 percent German- and 25 percent Italian-speaking inhabitants [8]. Different linguistic groups are served by the same national healthcare system and service provider. Linguistic group membership is an established determinant of health information-seeking behavior in South Tyrol [9].

Representative information on vaccination intention is important for an agile regional response to COVID-19 vaccination [10]. Several studies have investigated the causes of hesitant vaccination behavior in Italy and have described that among them, the individually different experiences of the pandemic with information overload and lifestyle changes played a significant role [11,12]. Using a representative survey of the inhabitants of South Tyrol, we examined demographic, linguistic, and social attitudes as well as COVID-19 health behavior to determine their correlation with hesitance to receive a COVID-19 vaccine in South Tyrol.

## 2. Materials and Methods

### 2.1. Study Design and Data Collection

This study used a cross-sectional, probability-based mode survey. The Provincial Institute for Statistics of the Autonomous Province of Bolzano–South Tyrol (ASTAT) recruited a random sample of citizens from South Tyrol using a strata-based sampling strategy by municipality, excluding nursing homes, sex, and age group (18–34, 35–49, 50–64, 65+ years), using the “Surveyselect” program in SAS v9.2. The participants were aged ≥ 18 years old. The survey was conducted in March 2021. ASTAT data management was performed in accordance with the General Data Protection Regulations of the EU.

More than 4000 of the 430,000 full-aged South Tyrolean inhabitants were invited to participate in the study following a one-stage random sampling design stratified for the quantitative study. The sample size was defined based on an expected participation rate of 33%, which was observed in previous surveys in this study series [13].

Participants were invited via a letter that included the planned participation date, a link to an online questionnaire (with telephone support) covering demographic, clinical, and socio-behavioral aspects, and a personalized password for use as a pseudo-anonymization code.

### 2.2. Questionnaire

The questionnaire was an extended version of the COSMO survey [14], and its repeated application was part of an Italian investigation. Questions regarding vaccine hesitancy were added to the official WHO questionnaire used in the COSMO (for a more detailed description of the questionnaire, see [15]). It contains instruments to measure trust in sources of information and institutions [16,17], conspiracy perceptions [18], resilience [19], and altruism [20].

Sociodemographic questions were adapted to the specific South Tyrolian situation, including items for the municipality as well as for the mother tongues of German, Italian, and Ladin. In addition, specific questions regarding social behavior and well-being were added [21].

### 2.3. Vaccine Hesitancy (Dependent Variable)

Vaccine hesitancy was measured using a dichotomous question: “Would you get vaccinated against COVID-19?”. To obtain more detailed information, questions about trust in vaccination, beliefs about the COVID-19 vaccine itself, and opinions regarding COVID-19 vaccination were added.

### 2.4. Putative Predictors of Vaccine Hesitancy (Independent Variables)

Sociodemographic variables were used to predict vaccine hesitancy. Information regarding age, gender, mother tongue (German, Italian, Ladin, other/more than one), residence, educational status on a 4-item scale, Italian citizenship, information about one’s living situation, healthcare profession, chronic diseases, and economic situation over the last 3 months as ranked on a 3-point scale that included the option “I don’t know” was asked for. Further, predictors for COVID-19 vaccination were taken from the literature and from the COSMO questionnaire: Trust in information sources and institutions [16,17] (health authorities and governments) was measured on a 6-point Likert scale ranging from 1 = “no trust” to 6 = “a lot of trust” (including a seventh “don’t know” response option). In addition, we measured conspiracy perceptions (5 questions on a 6-point Likert scale ranging from 1 = “don’t agree at all” to 6 = “completely agree”) [18], resilience (3 items on a 6-point Likert scale from 1 = “don’t agree at all” to 6 = “ completely agree”) [19], well-being within the last 2 weeks (5 items on a 4-point Likert scale from 1 = “always” to 4 = “never” [21]), and altruism (5 questions on a 6-point Likert scale from 1 = “don’t agree at all” to 6 = “completely agree”) [20]. All variables were considered predictors of vaccine hesitancy.

### 2.5. Statistical Analysis

Metric data are presented as the median and as the first and third quantiles due to the non-normality of all of the metric variables, and significant differences between groups were calculated using the Mann–Whitney U test. Nominal and ordinal data are presented as absolute numbers and percentages. The chi-square test and Cramér’s V were used to test for group differences, and Kendall’s Tau-b was used for correlations. 

Sum scores were calculated for conspiracy theories (Cronbach’s alpha = 0.81), well-being (Cronbach’s alpha = 0.88), resilience (Cronbach’s alpha = 0.66), altruism (Cronbach’s alpha = 0.79), trust in media (Cronbach’s alpha = 0.84), and trust in institutions (Cronbach’s alpha = 0.92). 

For trust in media and trust in institutions, the response option “I do not know” was coded once as 3.5 (mean of 1 to 6) and for the second time as “missing”. In the second approach, each sum score for each subject was only calculated when none of the questions were missing. A higher sum score indicated greater trust.

For comparisons of the sum scores regarding language-specific groups, we used the Kruskal–Wallis test with post hoc Mann–Whitney U tests for pairwise language comparisons. Post hoc tests were performed with Bonferroni correction for multiple testing.

Logistic regression was used to explain vaccine hesitancy based on the predictor variables. Significant and non-significant variables are presented. Metric variables were checked for linearity by testing the quadratic term for significance. Model diagnostics was performed using DFBETA statistics.

According to Bujang et al. [22], a minimum sample size of at least n = 500 is recommended for observational studies in large populations. Furthermore, it is suggested to adopt the formula n = 100 + i × 50, with “i” indicating the number of independent variables. Thus, with a total number of 22 independent variables in the main model (model 1), we achieved a total required sample size of n = 100 + 19 × 50 = 1050. Using classic sample size estimation, under the assumption of a basic probability of 15 percent and an odds ratio (OR) of 1.4 with a type one error of 5% and a power of 90%, the requested sample size was found to be n = 729. The sample size calculation was performed using G*Power version 3.1.9.4. *p*–values < 0.001 are indicated with ***, < 0.01 with **, and < 0.05, *, and *p*–values ≥ 0.05 are regarded as not significant (n.s.). All statistical analyses were performed using SPSS version 27.

## 3. Results

### 3.1. Sample Characteristics

A probability sample was drawn since extraction was made with a known probability from the population register. The sampling design is a one-stage stratified sampling plan with the stratification of individuals by municipality, gender, and age group. There were no a priori domains of study. The study universe consisted of the resident population, including immigrants. Cohabitation in residential facilities (nursing homes) was excluded. The estimates resulting from the survey were obtained using a calibration estimator. For this purpose, poststratification was applied with the known totals of the variables: age group, gender, municipality, and nationality. The response rate was 32%.

The demographic characteristics of the data set were representative of age, sex, and mother tongue. Table 1 presents sample characteristics across vaccine hesitancy groups. Participants with a chronic disease and older age were found to be significantly less hesitant than people without a chronic disease, and people with coronavirus infection were significantly more hesitant than people who had not yet been infected. Participants with children aged 0–6 years old were significantly more hesitant, and those living with a patient at risk of developing COVID-19 were significantly less hesitant. Participants with better economic status were significantly less hesitant. Urban residents were significantly less hesitant than rural residents, and significant differences were found in educational status and mother tongue. 

**Figure 1:**
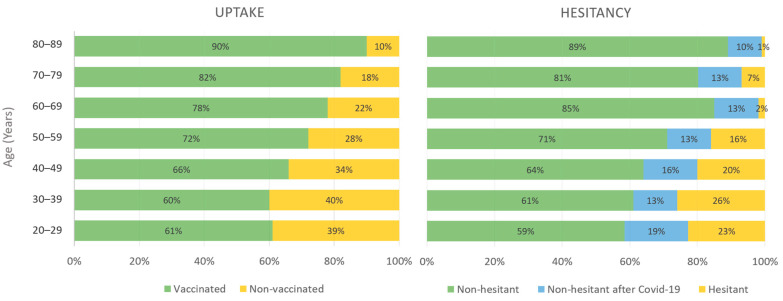
COVID-19 vaccine uptake on 6 September 2021 [23] versus COVID-19 vaccine hesitancy in March 2021 per age group in South Tyrol.

In our survey in March 2021, approximately 15% of the participants were in the hesitancy group. Hesitancy decreased significantly with increasing age (*p* *<* 0.001) (Figure 1). The percentage of people who were either vaccine non-hesitant or who had recovered from SARS-CoV-2 infection corresponded well with vaccine uptake until 6 September 2021 [23].

### 3.2. Vaccination Perception

#### 3.2.1. Compulsory Vaccination for Non-Coronaviruses

**Figure 2:**
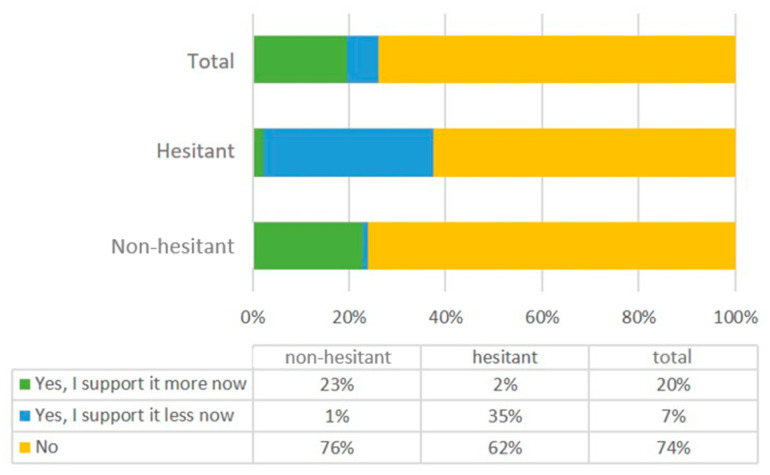
Participant responses to the question “Has the pandemic changed your attitude towards compulsory vaccination?”.

Participants were asked whether the pandemic changed their attitudes towards compulsory vaccination for non-coronaviruses. Almost three-quarters of the participants did not alter their attitudes towards compulsory vaccination for other viruses; one-fifth changed their attitudes to be more supportive of vaccinations because of the pandemic. This change was distributed differently among COVID-19 vaccination-hesitant and non-hesitant participants. Twenty-three percent of the non-hesitant participants stated that they supported mandatory vaccination for non-coronavirus diseases more, and 1% reported that they supported it less now; two percent of the non-hesitant participants supported it more, while 35% of them supported it less (Figure 2). An overall chi-square test detected a significant difference between the hesitant and non-hesitant participants (*p* < 0.001). Post hoc tests with Bonferroni correction resulted in highly significant outcomes for all items (No vs. Yes, I support it more now: *p* < 0.001; No vs. Yes, I support I less now: *p* < 0.001; Yes, I support it more now vs. Yes, I support it less now: *p* < 0.001).

#### 3.2.2. Vaccination Hesitancy and Attitudes towards COVID-19 Disease, Vaccines, and Vaccination

Trust in the system correlated significantly with non-hesitant participants (Table 2). Non-hesitant participants agreed significantly more with decisions about COVID-19 and COVID-19 vaccination made by the authorities. Trust in COVID-19 vaccination was highly correlated with confidence in vaccines (95%), while 60% of the hesitant participants did not trust vaccines. Trust in vaccine recommendations in general (95%) and recommendations made by healthcare personnel (93%) were highly correlated with confidence, while 16% and 17%, respectively, of the hesitant participants trusted these recommendations. In total, 27% of the non-hesitant and 75% of the hesitant participants would not get vaccinated if they knew that they had already been infected, while 8% and 53% of the non-hesitant and 53% of the hesitant participants thought that they did not have to get vaccinated if everyone else was vaccinated.

Additionally, 83% of non-hesitant participants agreed that everybody should be vaccinated according to the national vaccination plan, while 23% of the hesitant participants agreed. Less than 15% of the non-hesitant participants agreed with all of the statements that vaccination was unnecessary. It was found that 73% of the hesitant group believe that vaccination is only for the pharmaceutical industry to make a profit, 60% believe herd immunity is achieved with the spread of the virus, 55% believe that vaccination is not effective, and 34% believe that the virus does not exist/ is a normal flu.

Additionally, 91% of the hesitant participants think that vaccination is harmful because the long-term risks are not known, while 39% of the non-hesitant participants share this opinion. We also found that 73% of the hesitant participants but only 12% of the non-hesitant participants thought that new vaccines pose additional risks. Major socio-political discussions are expected by 67% of the hesitant and 30% of non-hesitant participants, and 65% of the hesitant and 14% of the non-hesitant participants mentioned that there are doctors who advise against COVID-19 infection. All of these variables are significantly correlated with vaccine hesitancy.

### 3.3. Predictors of Vaccine Hesitancy

Predictors and vaccine hesitancy were tested for significant associations (Table 3).

#### Trust in Information Sources

Trust in sources was significantly correlated with vaccine hesitancy. Non-hesitant participants trust media such as TV (38%), newspapers (27%), and radio (43%) significantly more than hesitant participants (9%, 11%, and 10%, respectively). Trust in healthcare workers was significantly different between non-hesitant (76%) and hesitant (44%) participants. No significant associations were found for trust in social media and trust in famous people/influencers.

The trust of non-hesitant participants in regional, national, and international health institutions ranged between 60% and 70%, while hesitant participants trusted these institutions significantly less (between 17% and 22%). Trust in regional civil protection was relatively high for both hesitant (72%) and non-hesitant (35%) groups, while trust in the regional government was lower at 58% and 19%, respectively. Hesitant participants (31%) searched for information about COVID-19 vaccination significantly less than non-hesitant participants (54%).

### 3.4. Specific Reasons for Vaccine Hesitancy: Total Scores

The results of the survey items on conspiracy perceptions, resilience, altruism, and well-being are also given in Table 3. Conspiracy perceptions were measured using five questions on a six-item scale. Each question was highly correlated with vaccine hesitance. Resilience was not significantly different between hesitant and non-hesitant individuals, and even the sum score did not result in a significant difference. Well-being was found to be significantly different only for the item “I feel fresh and relaxed”.

#### 3.4.1. Mistrust in Political and Scientific Authorities and Conspiracy Theories

For trust in media, a total score was calculated for TV, print media, social media, radio, and influencers. Total scores ranged from 5 to 30. Sum scores for trust in institutions were calculated for healthcare workers, the Ministry of Health, the National Institute of Health, the WHO, the regional toll-free and emergency number, civil protection, and the provincial government (ranging from 8 to 48). Sum scores for trust in media were significantly higher for non-hesitant participants (median (Q1;Q3), 13 (10;16)) than for hesitant participants (7 (5;11)), as was the sum score for trust in institutions (34 (27;40) and 19 (10;26), respectively).

For conspiracy theories, there were significantly higher scores in the calculated sum score for the hesitant respondents (median 21, [17;25]) than for the non-hesitant respondents (median 15 [11;20], *p* < 0.001).

#### 3.4.2. Altruism, Resilience, and Well-Being

For altruism, no significant difference was found between the hesitant and non-hesitant participants. For the item “I try to help others, even if they do not help me”, however, altruism was significantly different between the hesitant and non-hesitant participants (*p* < 0.05).

For resilience, the difference between the hesitant and non-hesitant participants was not significant (Table 3), and no significant difference was found between hesitant and non-hesitant participants for well-being.

### 3.5. Hesitancy-Related Attitudes in Mother Tongue Groups

Baseline characteristics among the participants of native language groups (German, Italian, Ladin, other language/ more than one language) differed with respect to age, gender, education level, Italian citizenship, urban vs. rural residence, the household characteristic “children from 0–6”, and economic situation in the last three months. Table 4 shows percentages for the hesitant and non-hesitant participants with respect to the formerly mentioned variables and language groups.

Comparing language groups for vaccine hesitancy, significant differences were found between German and “other/more than one” (*p* = 0.005) and Italian and “other/ more than one” (*p* < 0.001), respectively.

Analysis of specific age groups revealed that hesitancy was age-dependent and higher for young German-speaking participants than for young Italian participants (20–29 years: 25.8% vs. 9.3% and 30–39:29% vs. 17.9%) and was not significantly different for middle-aged German and Italian speakers (40–49 years: 17.6% vs. 16.2% and 50–59 years: 14.8% vs. 18.1%). No variation was observed for older age groups (all below 6% in hesitant participants). Data for the remaining language groups were too sparse to draw meaningful conclusions.

**Figure 3:**
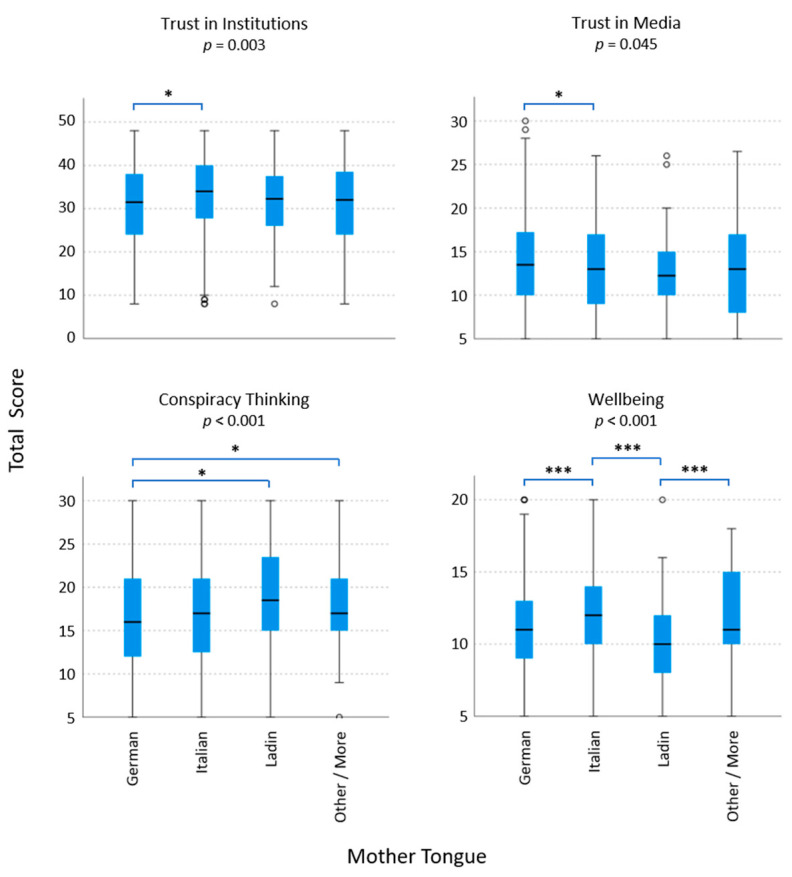
Boxplots of total scores for trust in institutions, trust in media, conspiracy thinking, and well-being of native language groups in South Tyrol during the pandemic. Differences between mother tongues were statistically analyzed in pairs and for multiple groups with pairwise Mann– Whitney tests and Bonferroni correction. For sum scores, see Methods. *, *p* < 0.05; ***, *p* < 0.001, and *p* < 0.05 without Bonferroni correction.

Native language groups differed in their trust in TV, press, social media, and radio as media information sources, and they differed in trust in all kinds of institutions involved in pandemic management, except for healthcare workers and the WHO. In addition, conspiracy theories and well-being scores were significantly different for language group memberships. Altruism and resilience scores did not show any language-specific differences, even though some single items resulted in significant differences between languages (data not shown). Figure 3 illustrates the significantly different sum scores for the mother tongues, including Bonferroni-adjusted pairwise comparisons. Trust in media was the lowest for participants speaking more than one language and the highest for participants speaking “another” language, while trust in institutions was the highest for participants speaking more than one language or Italian and the lowest for participants speaking German. Ladin speakers believed in conspiracy theories the most, and German speakers believed in them less; well-being was the lowest for the German and Ladin speakers and the highest for the Italian speakers (Figure 3).

**Figure 4:**
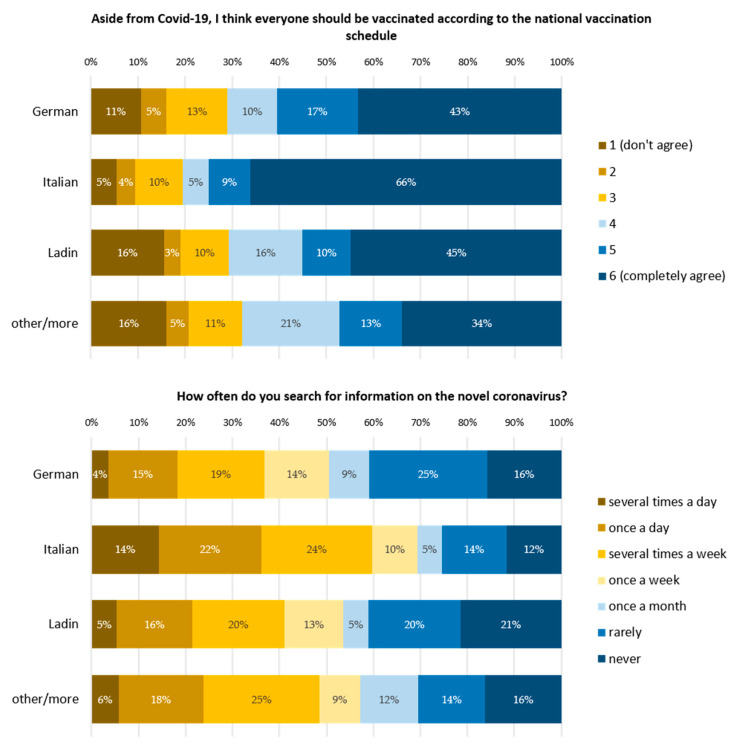
Attitudes towards national non-COVID-19 vaccination schedules (upper panel) and frequency of searching for coronavirus information related to native language group membership (lower panel). Upper panel: German vs. Italian, *p* < 0.001; Italian vs. other/more, *p* < 0.001; Italian vs. Ladin, *p* = 0.05; Italian vs. other/more, *p* < 0.001. Lower panel: German vs. Italian, *p* < 0.001; Italian vs. Ladin, *p* = 0.001; Italian vs. other/more, *p* = 0.028. P-values indicate Bonferroni-corrected significant differences from pairwise Mann–Whitney test after Kruskal–Wallis test (N = 1425). Both Kruskal–Wallis tests were significant, with *p* < 0.001.

Italian speakers agreed more with the national vaccination plan than all other language groups (Figure 4, upper panel: German vs. Italian, *p* < 0.001; Italian vs. Ladin, *p* = 0.009; Italian vs. other languages, *p* < 0.001; Italian vs. “more than one language”, *p* < 0.001; German vs. “more than one language, *p* = 0.047). Italian speakers searched for information about COVID-19 vaccination more frequently than all of the other language groups, and Germans showed the lowest frequency of searching for information. The frequency of searching for information on COVID-19 vaccines was found to be significantly different between some members of the various language groups (Figure 4, lower panel: German vs. Italian, *p* < 0.001; Italian vs. Ladin, *p* = 0.002).

### 3.6. Multivariable Logistic Regression to Predict Vaccine Hesitancy

A logistic regression model was used to identify independent predictors of vaccine hesitancy, including linguistic group membership (Italian as an indicator). Variables were significant demographic predictors and included age as a continuous factor, chronic disease, COVID-19 infection, urban residence, education, mother tongue, children aged 0–6 years or a patient at risk of COVID-19 in the family, and economic situation in the last three months. Further predictors included were the frequency of searching for information, altruism (helping others even when they do not help me), well-being (feeling fresh and awake), and general vaccine hesitancy. Total scores for conspiracy theories, trust in the media, and trust in institutions were also included.

A basic model (model 0) including only the demographic variables was calculated first and is presented in Table 5, as was the full model (model 1). To identify possible interactions, a further model (model 2) was added to the full model for the interaction terms “Italian mother tongue” *x* “trust in institutions”; “Italian mother tongue” *x* “Agree with the national vaccination plan”; “German mother tongue” *x* “search for information”; “German mother tongue” *x* “age”; and “Ladin mother tongue” *x* “conspiracy thinking”. Since all interaction terms were found to be not significant in model 2, details are not presented in Table 5. All models were tested for the linearity of metric predictors, including the corresponding quadratic terms. All quadratic terms were non-significant.

Models were calculated using N = 1425 cases and mean-adjusted sum scores. A recalculation of the models using only sum scores where all values were available (N = 864), as described in “Methods”, led to the same results (details not presented).

In model 0, age was confirmed as a significant decreasing predictor for vaccine hesitancy (*p* < 0.001, OR 0.963; 95% CI 0.953–0.973), as were a higher educational level (*p* = 0.001, OR 0.753; 0.641–0.884) and chronic disease (*p* = 0.005, OR 0.421; 0.229–0.771). Previous COVID-19 infection (*p* = 0.042, OR 1.454; 1.013–2.085) and a worse economic situation in the last three months (*p* = 0.004, OR 1.436; 1.124–1.836) were found to be significant increasing factors. Households with children aged 0–6 years old, households with patients at risk of developing severe COVID-19, and the participant himself/herself having previously been infected with COVID-19 were not significant factors in the model. Even urban residence and any single mother tongue (German, Italian, or Ladin) were not significant. The basic model (model 0) had Nagelkerkes R^2^=0.148.

Checking for pairwise correlations, Kendall’s Tau-b was significant for age and educational level (−0.272, *p* > 0.01); age and chronic disease (0.270, *p* < 0.01); age and a worsening economic situation (−0.057, *p* < 0.05); age and Italian as a mother tongue (0.076, *p* < 0.01); higher educational level and chronic disease (−0.159, *p* < 0.01); higher educational level and urban residency (0.106, *p* < 0.01); higher educational level and German as a mother tongue (−0.103, *p* < 0.01); higher educational level and Italian as a mother tongue (0.074, *p* < 0.01); chronic disease with lower educational level (0.056, *p* < 0.05); worsening economic situation with urban residency (−0.072, *p* < 0.05); Ladin language (0.089, *p* < 0.01) and Italian language (−0.057, *p* < 0.05); and urban residency with German (−0.440, *p* < 0.01), Italian (−0.483, *p* < 0.01), and Ladin (0.127, *p* < 0.01) as a mother tongue. Excluding the variables chronic disease and urban/rural residency from the model did not change the results or lead to a better model fit.

In model 1, age was confirmed as a significant decreasing predictor for vaccine hesitancy (OR 0.966; 95% CI 0.952–0.980], as was chronic disease (OR 0.356; 0.159–0.795). A high educational level had a significant negative effect (OR 0.785; 0.642–0.988) on vaccine hesitance. Lower frequencies of searching for information regarding COVID-19 vaccination (OR 1.234; 1.093–1.393) significantly contributed to vaccine hesitancy. Agreement with the general national vaccination plan (OR 0.445; 0.399–0.520) significantly decreased the risk of vaccine hesitancy. Finally, believing in conspiracy theories significantly increased (OR 1.113; 1.071–1.157) and trust in institutions significantly decreased the risk of vaccine hesitancy (OR 0.936; 0.904–0.965).

Altruism (“to help others, even when they do not help me”) and feeling fresh and awake did not exert a significant effect in the regression model, nor did trust in the media. The logistic regression model had an overall Nagelkerkes R^2^ of 0.61, and an overall model quality of 92% was estimated using ROC analysis (area under the curve: 0.935; 95% CI 0.928–0.972).

A worse economic situation and former COVID-19 infection, both of which were significant in model 0 (*p* = 0.004 and *p* = 0.042, respectively), were not more significant in model 1.

Analysing the economic situation for correlations with the model terms, a negative correlation worsened with trust in the national vaccination plan (−0.069, *p* < 0.01), trust in media (−0.124, *p* < 0.05), and trust in institutions (−0.123, *p* < 0.05), and there was a positive correlation with belief in conspiracy theories (0.104, *p* < 0.01). The three variables of trust and the belief in conspiracy theories are correlated between each other (trust in the national vaccination plan with trust in media: 0.180, *p* < 0.01; trust in the national vaccination plan with trust in institutions: 0.329, *p* <0.01; trust in the national vaccination plan with conspiracy theories: −0.156, *p* <0.01; trust in media with trust in institutions: 0.415, *p* < 0.01; trust in media with conspiracy theories: -0.130, *p* < 0.01; trust in institutions with conspiracy theories: −0.211, *p* < 0.01), but excluding any of them or combinations of them did not change the significance of the other variables, nor did it lead to a better model fit.

Model diagnostics with DFBETA statistics showed that the model was stable and did not change when excluding single cases.

## 4. Discussion

In our study of a probability-based sample of 1425 citizens form the Autonomous Province of Bolzano, Italy, which was conducted with the aim of identifying possible South Tyrol-specific predictors of COVID-19 vaccine hesitancy in March 2021, 15.6% of respondents reported being hesitant, a proportion similar to the reported 18.1% vaccine hesitancy determined in a population-based, social media-recruited survey performed in Italy in December 2020 [24]. A representative survey performed in the Italian region of Reggio-Emilia reported vaccine hesitancy at a rate of 31.1% at the beginning of the European vaccination campaign in January 2021 [25]. Changes in vaccine hesitancy rates over time have been noted, complicating quantitative comparisons [26]. Levels of volatility around vaccine hesitancy have been well-documented by several studies and are related to factors including new information, new policies, or newly reported vaccine risks as well as to the public’s trust in experts, preferences for alternative health, political polarization, and belief-based extremism [10]. As with disparities in COVID-19 disease severity, countries with high pandemic vaccination coverage attributed the greater hesitancy among ethnic minorities to lower social status and fewer social assets compared to predominant populations [27]. In our study, mother tongue group membership was correlated with hesitancy in addition to younger age, lower educational level, pandemic-related worsening of one’s economic situation, the absence of comorbidities, not yet having been infected with SARS-CoV-2-, having children 0–6 years of age, no patient at risk of COVID-19 living in the same household, and rural residence. This identification of predictors confirms, to a large extent, previous observations [10,24,25]. However, to our knowledge, the role of linguistic group membership in COVID-19 vaccine hesitancy has not yet been previously reported in a representative population-based survey.

Guatemalans speak 25 languages in various dialects, yet educational material promoting COVID-19 vaccines were at first published exclusively in Spanish, and this is now being held responsible for the failure of the COVID-19 vaccine roll-out [28]. Given mistrust as a frequent factor in the literature regarding COVID-19 vaccine hesitancy in visible minority populations [28,29,30], a newspaper article related the pandemic situation to the suggestion, among others, of historical mistrust in South Tyrol against the central Italian government [31]. In December 2021, the weekly incidence rates of the coronavirus pandemic put South Tyrol were among the worst of all Italian regions, and the vaccination rate was the lowest in Italy, although public healthcare quality is considered to be above average [32]. While the majority in South Tyrol are German-speaking, Italian speakers tended to be more obedient during the coronavirus pandemic. Correspondingly, 20% of the school staff at German-language schools were unvaccinated compared to 3 percent in Italian schools [33]. German speakers in South Tyrol seem to have lower levels of trust in health authorities. A survey in May 2021 found that 44% of German speakers trusted the Italian National Institute of Health pandemic recommendations compared to 70% of Italian speakers [33].

South Tyrol had low levels of childhood vaccination before the coronavirus pandemic, with only 71.9% of children aged 24 months being jabbed against measles in 2017 instead of the average range of 90% [34]. Web-based information programs have already been launched at the national level in Italy in 2013 to improve vaccination behavior and regional disparities [35], with varying success [36]. A survey conducted in 2017 in the South Tyrolean population (N = 200) asking about the vaccine hesitancy of parents regarding compulsory vaccination according to a new national vaccination plan revealed an overall vaccine hesitancy of parents of about 29% and a low information standard of about 40% [37]. A clear difference in the hesitancy between the German (35%) and Italian (15%) participants was found. This result could not be verified in this study regarding COVID-19 vaccine hesitancy. An overall hesitancy of about 15% in the full-aged population was observed, but the difference in hesitance between German (84.2%) and Italian (88.3%) participants was only about 4%. In our data, differences in vaccine hesitancy for COVID-19 between German and Italian participants were found for the groups of 20–29 and 30–39. This corresponds in part to the results of Atz [37] but does not explain the overall vaccine hesitancy in South Tyrol.

Comparing the vaccine hesitancy results of South Tyrol in March 2021 with the COVID-19-vaccination rates in South Tyrol observed in September 2021 [23], before compulsory COVID-19 vaccination started in Italy for special population groups, 32% of people aged 19 years old or above had not yet received their first vaccine dose in South Tyrol. Assuming that people with an infection did not receive the vaccine at that time according to the vaccination strategy, we calculated that the number of people who were either hesitant or infected and reached 29%. The difference between the true vaccination rate and the rate of people either infected or hesitant until April 2021 was less than 4% in all age groups, except for the age group of participants aged between 60 and 69 years old (difference of 7%), confirming that hesitancy explains a significant proportion of non-vaccination at that time.

A comparison of language groups clearly showed an elevated mistrust in institutions in the German and Ladin populations of South Tyrol and an elevated trust in conspiracy theories in the Ladin population. Mistrust in institutions and conspiracy theories have both been identified as important determinants of vaccine hesitancy and resistance [38,39]. Furthermore, well-being, which is associated with higher vaccine acceptance [27], was lowest in the German and Ladin populations and was the highest in Italians. However, these results cannot be adopted directly due to vaccine hesitance. Well-being was not found to be an indicator of vaccine hesitance, while mistrust in institutions was found to be one. Demographic variables showing significant differences between language groups failed to correlate significantly with native languages. Only high educational status was found to have a positive effect on trust in vaccination, and Italian participants were found to have an elevated educational status. Ladin speakers were found to be more hesitant, which may be more closely related to changes in their economic situations. However, this result did not appear to affect vaccine hesitancy.

All of these findings are consistent with the fact that compared to other languages, native Italian speakers gave the highest approval to the national vaccination recommendations for COVID-19 and also had the highest interest in information about the pandemic (Figure 4). How good the knowledge about the health benefits of vaccination and its association with trust are in the population of South Tyrol was not explicitly investigated in the study. Quantitative survey results for health literacy are not available. Information-seeking behavior, which is a determinant of health literacy, was studied population-wise before the pandemic and was described as differing between language groups [9]. In the present study, members of the Italian language group with lower vaccine hesitancy sought information about the pandemic more frequently than members of the German language group.

The observed differences in vaccine hesitancy between the three main language groups in South Tyrol were minor (Italian, 88%; German, 84%; Ladin 83%). Predictor variables such as trust in institutions, frequency of searching for information, and trust in the national vaccination plan were correlated with languages, especially the Italian language, but no language effect was found in the regression analyses, neither for the language alone, nor for the interaction of language with any of the variables. Thus, we can conclude that predictor variables correlate with language, but the effect of language is not strong enough to impact COVID-19 vaccine hesitancy. All variables regarding trust and conspiracy thinking correlate with each other as well as with the worsening economic situation variable. These variables have the greatest impact on the model as an explanation of vaccine hesitancy.

Since the interaction terms of the variables regarding trust and conspiracy theories with languages were found to be not significant, once more, the language effects cannot be identified as strong predictors. However, we have found that conspiracy theories are especially prevalent in the Ladin-speaking population, mistrust in institutions is prevalent in the German-speaking population, mistrust in media is prevalent in the Italian population, and mistrust in the general national vaccination plan is prevalent in the German- and Ladin-speaking population. Thus, general mistrust, which explains the vaccine hesitancy in South Tyrol the best, concerns different language groups in different ways. Thus, strategies to increase trust differently according to language group could be developed.

A direct comparison of hesitant vaccination behavior during the coronavirus pandemic in neighboring countries has been made between Ireland and the United Kingdom, and it has been shown that psychological constructs are less crucial than sociodemographic and health-related factors [40]. As languages also correlate with economic status, we can assume that minority groups other than the visible Italian and German speakers may be marginalized communities in South Tyrol, in whom increased vaccine hesitancy may arise, as seen in other parts of the world [6,7,28].

Italy adopted a mandatory vaccination policy before the pandemic to achieve target immunization coverage rates and to limit outbreaks. In July 2017, the Italian Ministry of Health approved law no. 119, which extended the number of mandatory vaccinations for school attendance from four to ten [41]. Vaccine hesitancy for mandatory vaccinations is higher in Trentino–Alto Adige than in other regions of Italy [42], and vaccine uptake in South Tyrol has continued to remain below the Italian average [43]. Considering the overall positive effect of the introduction of child vaccine mandates [44,45], Italy extrapolated this policy to COVID-19 vaccination [46]. The results of the present study suggest that linguistic group membership does not directly impact the vaccine hesitancy of the three main languages sponsored in South Tyrol and are hence unlikely to interfere with national vaccination plans beyond associated sociodemographic and cultural predictors.

The limitations of the study include unmeasured variables of potential importance for vaccine hesitancy. Although this study collected data on whether the pandemic led to COVID-19 disease or the infection of loved ones, data on lifestyle changes due to the pandemic measures and knowledge of the disease as discussed by Gallè and co-workers [11] were poorly examined, so their influence on vaccination behavior could not be directly analyzed. Other variables include political orientation or the use of alternative and complementary medicine, which are known predictors [47,48,49]. In 2014, in South Tyrol, awareness and use of home remedies were investigated in a population-based cross-sectional telephone survey, and the results indicated that linguistic group membership in South Tyrol was associated with awareness of home remedies and their use in that pre-pandemic study [50]. Whether this finding may affect the relationship between linguistic group membership and vaccine hesitancy remains unknown. In addition, if the findings presented here on the linguistic correlates of vaccine hesitancy from the second year of the pandemic are valid in later periods [10] is another limitation. Finally, children and adolescents were excluded from the survey, leaving their impact on potentially important predictors of vaccine hesitancy in the context of language group membership and minorities uninvestigated, even though the vaccination of children and adolescents has become increasingly important during the course of the pandemic.

## 5. Conclusions

In the second year of the pandemic, COVID-19 vaccine hesitancy was higher in German speakers than in the native Italian language group of South Tyrol, whose health service is part of the national Italian healthcare system. Whether the difference is partly related to linguistic group membership and the historically negative attitudes of German speakers towards the national central governance of the peripheral autonomous province by Rome has been analyzed in this probability-based survey. The results suggest that the greater vaccine hesitancy in the German language group in South Tyrol can be sufficiently explained by other variables known to reduce vaccine acceptance, which are distributed differently among the language groups. This finding may inform health policies and vaccination campaigns to increase vaccine acceptance.

## Figures and Tables

**Table 1:** Characteristics of the sample and comparison between non-hesitant and hesitant individuals.

Characteristics N (%)	Overall 1425 (100) N (%)	Non-Hesitant 1204 (84.4) N (%)	Hesitant 222 (15.6) N (%)	*p*–Values ^†^
Age (years)				<0.001
18–34	334 (23.4)	248 (74.3)	86 (25.7)	
35–49	354 (24.8)	281 (79.4)	73 (20.6)	
50–64	391 (27.4)	342 (87.5)	49 (12.5)	
≥64	346 (24.3)	332 (96)	14 (4.0)	
Gender				n.s.
Male	691 (48.5)	587 (84.9)	104 (15.1)	
Female	735 (51.5)	617 (83.9)	118 (16.1)	
Education				0.001
Middle school or lower	316 (22.2)	278 (88)	38 (12)	
Vocational school	411 (28.9)	335 (81.5)	76 (18.5)	
High school	410 (28.8)	332 (81.0)	78 (19)	
University	287 (20.2)	258 (89.9)	29 (10.1)	
Residence				0.013
Urban	602 (42.2)	525 (87.2)	77 (12.8)	
Rural	824 (57.8)	679 (82.4)	145 (17.6)	
Citizenship				n.s.
Italian	1307 (91.7)	1107 (84.7)	200 (15.3)	
Other	118 (8.3)	96 (81.4)	22 (18.6)	
Native Language ^‡^				<0.001
German	879 (61.7)	740 (84.2)	139 (15.8)	
Italian	384 (26.9)	339 (88.3)	45 (11.7)	
Ladin	57 (4)	47 (82.5)	10 (17.5)	
Other/more than one	106 (7.3)	78 ()	28 ()	
Household/Family structure (more than one answer possible)				
Single	236 (16.5)	206 (87.3)	30 (12.7)	n.s.
Children 0–6 years of age	178 (12.5)	137 (77.0)	41 (23.0)	0.003
Adolescents 7–17 years of age	276 (19.4)	223 (85.4)	168 (14.6)	n.s.
Patient at risk of COVID-19 ^+^	301 (21.1)	275 (91.4)	26 (8.6)	<0.001
None of the above	522 (36.6)	432 (82.8)	90 (17.2)	n.s.
Working in the health sector				n.s.
Yes	85 (6.0)	76 (89.4)	9 (10.6)	
No	1340 (94.0)	1127 (84.4)	222 (15.6)	
Chronic disease(s)				<0.001
Yes	246 (17.3)	233 (94.7)	13 (5.3)	
No	1179 (82.7)	970 (82.3)	209 (17.7)	
COVID-19 infected				0.003
Yes	251 (17.6)	197 (78.5)	54 (21.5)	
No	1173 (82.4)	1006 (85.8)	167 (14.2)	
Economic situation (last 3 months)				<0.001
Better	43 (3.0)	43 (100)	0 (0)	
The same	972 (68.3)	850 (87.4)	122 (12.6)	
Worse	375 (26.3)	285 (76.0)	90 (24.0)	
Don’t know	34 (2.4)	25 (73.5)	9 (26.5)	

^†^ *p*-values refer to chi-square tests for non-hesitant vs. hesitant. ^‡^ Mother tongue of South Tyrolean inhabitants. ^+^ Participants living together with a patient at risk of COVID-19 in the same household.

**Table 2:** Attitudes towards COVID-19 disease, vaccines, and vaccination of vaccination non-hesitant vs. hesitant survey participants.

Category	Question	Response (Rather) Agree	Total N = 1425 (100%) N (%)	Non-Hesitant ^1^ N = 1204 (84.4%) N (%)	Hesitant ^1^ N = 222 (15.6%) N (%)	Cramér’s V (*p*–Value)
Decision making	I think that decisions about COVID-19 made by the public authorities are right	Yes	788 (55)	761 (97)	27 (3)	0.489 (<0.001)
		No	575 (40)	393 (68)	182 (32)	
	I think that decisions about vaccination against COVID-19 made by the public authorities are right	Yes	992 (70)	957 (96)	35 (4)	0.637 (<0.001)
		No	331 (23)	162 (49)	169 (51)	
	I think that decisions about compulsory vaccination (not COVID-19) made by the public authorities are right	Yes	952 (67)	924 (97)	28 (3)	0.658 (<0.001)
		No	348 (24)	170 (49)	178 (51)	
Trust in COVID-19 vaccination ^2^	I believe vaccination can contain the spread of the virus ^1^	Yes	1227 (86)	1139 (93)	89 (7)	0.65 (<0.001)
		No	198 (14)	65 (33)	133 (67)	
	If I knew that I’d already been infected with the virus, I wouldn’t get vaccinated	Yes	492 (34)	326 (66)	166 (34)	0.44 (<0.001)
		No	933 (65)	877 (94)	56 (6)	
	When others are vaccinated against the virus, I don’t need to get vaccinated	Yes	220 (15)	101 (6)	119 (54)	0.557 (<0.001)
		No	1206 (85)	1102 (92)	103 (8)	
	If vaccination was recommended to me, I would get vaccinated	Yes	1174 (82)	1138 (97)	36 (3)	0.789 (<0.001)
		No	251 (18)	66 (26)	185 (74)	
	If my doctor recommended COVID-19 vaccination, I would get vaccinated	Yes	1153 (81)	1116 (97)	37 (3)	0.768 (<0.001)
		No	273 (19)	88 (32)	185 (68)	
COVID-19 vaccination is not necessary because…	It is not effective	Yes	197 (14)	76 (39)	121 (61)	0.577 (<0.001)
		No	1229 (86)	1128 (92)	101 (8)	
	…natural herd immunity is achieved with virus spread and that is quite sufficient	Yes	274 (19)	141 (12)	133 (60)	0.512 (<0.001)
		No	1151 (81)	1063 (92)	89 (8)	
	…this disease does not exist/is a normal flu	Yes	150 (10)	74 (6)	76 (34)	0.44 (<0.001)
		No	1276 (90)	1130 (89)	146 (11)	
	…the whole thing is only a profit for the pharmaceutical industry	Yes	321 (22)	159 (49)	162 (51)	0.583 (<0.001)
		No	1105 (78)	1045 (95)	60 (5)	
COVID-19 vaccination is harmful because…	...long-term risks are not known	Yes	665 (47)	465 (70)	201 (30)	0.516 (<0.001)
		No	760 (53)	740 (97)	20 (3)	
	…new vaccines pose additional risks in the RNA	Yes	306 (21)	147 (48)	159 (52)	0.571 (<0.001)
		No	1119 (79)	1056 (94)	63 (6)	
	...there are doctors who advise against it	Yes	308 (22)	163 (53)	145 (47)	0.497 (0.001)
		No	1117 (78)	1040 (93)	77 (7)	
	…a compulsory corona vaccination with the prioritization of certain groups will lead to major socio-political discussions	Yes	512 (36)	366 (30)	148 (67)	0.33 (<0.001)
		No	912 (64)	838 (92)	74 (8)	

^1^ Vaccine hesitancy was measured with a dichotomous question: “Would you get vaccinated against COVID-19?”. ^2^ *p*-values refer to the chi-square test.

**Table 3:** Variables related to vaccine hesitancy and the effects of linguistic group membership in South Tyrol, Italy.

Variable ^1^	Question	Non-Hesitant ^3^ N = 1204 N (%)		Hesitant ^3^ N = 222 N (%)		Cramér’s V (*p*–Value) ^2^
		More Than Once a Week		More Than Once a Week		
Frequency of searching for information on COVID-19	How often do you search for information on the novel coronavirus?	586 (49)		42 (19)		0.323 ***
		(Rather) trust/agree	Do not know	(Rather) trust/agree	Do not know	
General national vaccination schedule	Aside from COVID-19, I think everyone should be vaccinated according to the national vaccination schedule	993 (83)	n.a.	51 (23)	n.a.	0.593 ***
Trust—How much do you trust information about COVID-19 and vaccinations from the following sources?	TV	455 (38)	56 (4.7)	19 (9)	5 (2.4)	0.406 ***
	Newspapers/press	443 (27)	55 (4.5)	24 (11)	11 (5.2)	0.354 ***
	Healthcare workers	911 (76)	57 (4.8)	98 (44)	13 (6)	0.365 ***
	Social media	144 (12)	122 (10.1)	18 (8)	12 (5.3)	0.143 ***
	Radio	512 (43)	100 (8.3)	23 (10)	13 (5.9)	0.383 ***
	Ministry of Health	788 (65)	84 (7)	49 (22)	18 (7.9)	0.441 ***
	National Institute of Health	695 (68)	201 (16.7)	37 (17)	35 (15.6)	0.442 ***
	Famous people and influencers	138 (11)	152 (12.6)	6 (3)	19 (8.5)	0.166 ***
	WHO	774 (64)	77 (6.4)	48 (22)	16 (7.1)	0.441 ***
	Regional toll-free and emergency numbers	568 (47)	338 (28.1)	48 (2%)	53 (23.7)	0.341 ***
	Civil protection	863 (72)	104 (8.6)	77 (35)	23 (10.3)	0.423 ***
	Provincial government	701 (58)	42 (35)	42 (19)	10 (4.7)	0.419 ***
	Management of the South Tyrolean Health Service	776 (64)	41 (3.4)	48 (22)	11 (4.9)	0.386 ***
Conspiracy—CMQ (Conspiracy Mental Questionnaire)	I think that many very important things happen in the world that the public is never informed about	765 (64)	n.a.	187 (84)	n.a.	0.241 ***
	I think politicians usually do not tell us the true motives for their decisions	627 (52)	n.a.	177 (80)	n.a.	0.308 ***
	I think that government agencies closely monitor all citizens	315 (26)	n.a.	92 (41)	n.a.	0.206 ***
	I think that events that superficially seem to lack a connection are often the result of secret activities	320 (27)	n.a.	105 (48)	n.a.	0.222 ***
	I think that there are secret organizations that greatly influence political decisions	383 (32)	n.a.	126 (57)	n.a.	0.258 ***
Resilience	I have a hard time making it through stressful events	375 (31)	n.a.	71 (32)	n.a.	0.044 (n.s.)
	It does not take me long to recover from a stressful event	628 (52)	n.a.	123 (56)	n.a.	0.083 (n.s.)
	It is hard for me to snap back when something bad happens	419 (35)	n.a.	79 (36)	n.a.	0.08 (n.s.)
Altruism—Altruistic Attitudes Among Older Adults Scale	I enjoy doing things for others	966 (80)	n.a.	180 (81)	n.a.	0.03 (n.s.)
	I try to help others, even if they do not help me	933 (77)	n.a.	152 (68)	n.a.	0.098 *
	Seeing others prosper makes me happy	1032 (86)	n.a.	189 (85)	n.a.	0.074 (n.s.)
	I really care about the needs of other people	903 (75%)	n.a.	165 (75)	n.a.	0.073 (n.s.)
	I come first and should not have to care so much for others	187 (16%)	n.a.	36 (16)	n.a.	0.032 (n.s.)
Well-being—In the last two weeks … ^4^	…I was happy and in a good mood	797 (66)	n.a.	138 (62)	n.a.	0.079 (n.s.)
	…I was calm and relaxed	802 (67)	n.a.	134 (60)	n.a.	0.052 (n.s.)
	…I was active and energetic	705 (59)	n.a.	131 (59)	n.a.	0.042 (n.s.)
	…I woke up fresh and rested	742 (62)	n.a.	124 (56)	n.a.	0.094 **
	…my everyday life was full of things that interest me	688 (57)	n.a.	109 (49)	n.a.	0.072 (n.s.)

^1^ Variables that are supposed to have an effect on vaccine hesitancy. ^2^ *p*–values refer to Pearson’s chi-squared test. Cramér’s V is computed by dividing the square root of the chi-squared statistic by the sample size and the minimum dimension minus one. ^3^ Vaccine hesitancy was measured with a dichotomous question: “Would you get vaccinated against COVID-19?”. *, *p* < 0.05; **, *p* < 0.01; ***, *p* < 0.001; n.s., not significant; n.a., not applicable.

**Table 4:** Language-specific baseline characteristics and vaccine hesitancy of study participants.

Characteristic		Hesitancy	German	Italian	Ladin	Other/More Than One	*p*-Value *
			N = 879 %	N = 384 %	N = 57 %	N = 106	
Age (median (IQR))		No	53 (38;67)	56 (40;70)	54 (44;64)	38 (30;51)	<0.001
		Yes	36 (28;49)	47 (35;54)	44 (31;50)	43 (30;48)	
Females (%)		No	54.6	44.1	42.6	56.4	0.001
		Yes	55.4	37.8	70.0	64.3	
Education (%)	Middle school or lower	No	23.2	23.3	19.1	23.1	<0.001
		Yes	15.8	15.6	10.0	32.1	
	Vocational school	No	32.8	18.9	34.0	16.7	
		Yes	41.0	24.4	60.0	7.1	
	High school	No	25.9	30.1	29.8	32.1	
		Yes	35.3	37.8	10.0	39.3	
	University	No	18.1	27.7	17.0	28.2	
		Yes	7.9	22.2	20.0	21.4	
Italian citizenship (%)		No	95.4	97.6	100.0	29.9	<0.001
		Yes	98.6	91.1	100.0	39.3	
Household / family structure (%) ^†^	Single	No	18.5	16.5	8.5	11.7	n.s.
		Yes	14.4	13.3	10.0	4.3	
	Children from 0–6	No	11.9	8.3	8.3	22.1	0.001
		Yes	20.1	11.1	10.0	25.0	
	Adolescents from 7–17	No	19.2	16.5	10.4	26.9	n.s.
		Yes	21.6	22.2	40.0	32.1	
	Patient at risk of COVID-19	No	23.4	23.1	25.5	15.4	n.s.
		Yes	13.8	11.1	10.0	3.6	
	None of the above mentioned	No	34.9	38.3	50.0	26.9	n.s.
		Yes	39.9	44.4	44.4	35.7	
Working in the health sector (%)		No	6.6	5.9	8.3	5.1	n.s.
		Yes	2.9	2.2	0	10.7	
Chronic disease(s) (%)		No	19.5	19.8	17.0	17.9	n.s.
		Yes	7.9	4.4	0	0	
Economic situation of the last 3 months (%)	Better	No	3.4	3.8	6.4	2.6	<0.001
		Yes	0	0	0	0	
	The same	No	72.3	76.4	42.6	46.2	
		Yes	54.7	71.1	50.0	35.7	
	Worse	No	22.0	18.9	51.1	44.9	
		Yes	41.7	24.4	50.0	53.6	
	Don’t know	No	2.3	0.9	0	6.4	
		Yes	3.6	4.4	0	10.7	
COVID-19 infected (%)		No	16.5	16.8	22.9	10.4	n.s.
		Yes	29.0	11.1	20.0	25.0	
Urban residence (%)		No	26.1	82.2	12.8	61.0	<0.001
		Yes	19.4	77.8	10.0	50.0	
Hesitancy (%)		No	26.1	82.2	12.8	61.0	0.002
		Yes	19.4	77.8	10.0	50.0	
Conspiracy thinking (median (IQR))		No	15 (11;19)	16 (11;20)	18 (14;23)	16 (15;20)	<0.001
		Yes	21 (16;24)	23 (19;27)	20 (19;27)	20 (18;24)	
Trust in institutions (median (IQR))		No	33 (27;38)	35 (30;40)	35 (29;39)	35 (27;43)	0.003
		Yes	20 (12;28)	23 (12;30)	27 (20;32)	21 (13;28)	
Trust in media (median (IQR))		No	14 (11;18)	13 (10;17)	12 (10;15)	14 (11;19)	0.045
		Yes	8 (5;13)	9 (6;13)	13 (10;16)	7 (5;10)	
Trust in the national vaccination plan (median (IQR))		No	6 (4;6)	6 (5;6)	6 (4;6)	5 (4;6)	<0.001
		Yes	2 (1;4)	2 (1;3)	2 (1;4)	3 (1;4)	
Frequency of information search (median (IQR))		No	3 (2;5)	4 (3;6)	4 (2;6)	3 (2;5)	<0.001
		Yes	6 (2;7)	6 (6;7)	6 (6;7)	6 (3;6)	

* Overall chi-square test for categorical data and Kruskal–Wallis test for metric data ^†^ More than one possible answer.

**Table 5:** Predictors of vaccination hesitancy in South Tyrol, Italy, in March 2021 in multivariate logistic regression analysis.

		Model 0 Nagelkerkes R^2^ = 0.614			Model 1 Nagelkerkes R^2^ = 0.614		
		Regression Coefficient B	*p*-Value	OR (95% CI)	Regression Coefficient B	*p*-Value	OR (95% CI)
Constant term		0.293	n.s.	1340	2475	0.035	11,879
Age		−0.038	0.000	0.963 (0.963;0.973)	–0.035	0.000	0.966 (0.952;0.980)
Educational level ^#^		−0.284	0.001	0.753 (0.641;0.884)	–0.241	0.039	0.785 (0.624;0.988)
Family and risk patterns	Children 0–6 years	0.089	n.s.	1.093 (0.728;1.641)	0.352	n.s.	1.422 (0.819;2.470)
	Patient at risk of COVID-19	−0.392	n.s.	0.675 (0.429;1.064)	–0.588	n.s.	0.555 (0.289;1.068)
	Chronic disease	−0.866	0.005	0.421 (0.229;0.771)	–1.033	0.012	0.356 (0.159;0.795)
	COVID-19 infected	0.374	0.042	1.454 (1.013;2.085)	0.227	n.s.	1.255 (0.769;2.048)
Search for information					0.210	0.001	1.234 (1.093;1.393)
Altruism	I try to help others, even when they do not help me				0.046	n.s.	1.047 (0.889;1.234)
Well-being	Feeling fresh an awake				0.099	n.s.	1.104 (0.871;1.399)
Trust	Agree with national vaccination plan				–0.787	0.000	0.455 (0.399;0.520)
	Trust in media				–0.024	n.s.	0.976 (0.918;1.037)
	Trust in institutions				–0.066	0.000	0.936 (0.907;0.966)
Urban		−0.236	n.s.	0.790 (0.548;1.138)	0.045	n.s.	1.046 (0.621;1.761)
Mother tongue	Italian ^†^		n.s.			n.s.	
	German	0.000	n.s.	1.000 (0.652;1.533)	–0.623	n.s.	0.536 (0.286;1.005)
	Ladin	0.025	n.s.	1.025 (0.447;2.349)	–0.515	n.s.	0.597 (0.197;1.811)
	Other/more than one	0.457	n.s.	1.580 (0.891;2.801)	–0.061	n.s.	0.941 (0.431;2.053)
Economic situation ^#^		0.362	0.004	1.436 (1.124;1.836)	0.042	n.s.	1.043 (0.770;1.413)
Conspiracy thinking					0.107	0.000	1.113 (1.071;1.157)

*p*-values for the significant contribution of the independent variables to the model. ^#^ Only one answer possible, categorical variable ^†^ Used as indicator.

## Data Availability

The data presented in this study are available upon request from the corresponding author. The data are not publicly available for political reasons such as conspiracies and ethno-linguistic content.

## Supplementary Materials

The following supporting information can be downloaded from https://www.mdpi.com/article/10.3390/vaccines10101584/s1, Table S1: SARS-CoV-2 vaccination rates (percent of population) in Italian regions and provinces on 17 January 2022. Table S2: SARS-CoV-2 vaccination rates (percent of population) in countries neighboring Italy in Central Europe on 13 January 2022.
