# Supplementary material for: Vaccine Hesitancy during the Coronavirus Pandemic in South Tyrol, Italy: Linguistic Correlates in a Representative Cross-Sectional Survey
Source: Vaccines (Basel). 2022 Sep 21;10(10):1584. doi: 10.3390/vaccines10101584 (PMC9607221; doi:10.3390/vaccines10101584)
Supplement: Supplementary file 1 [file vaccines-10-01584-s001.zip › vaccines-1879895-supplementary.pdf]

Barbieri et al.

Vaccines 2022

Supplementary Material

Table S1. SARS-CoV-2 vaccination rates (percent of population) in Italian regions and provinces on January 17, 2022[1].

Italian Region	First Dose[2] (%)	Full (%)	Booster (%)
Abruzzo	78.7	73.8	44.7
Basilicata	79.8	73.6	43.9
Bolzano	74.2	67.0	41.3
Calabria	76.5	68.6	41.1
Campania	76.3	69.6	42.7
Emilia-Romagna	80.4	74.6	45.6
Friuli – Venezia Giulia	76.9	71.4	39.8
Lazio	80.7	72.0	48.1
Liguria	79.6	74.4	40.9
Lombardia	81.9	75.2	48.2
Marche	77.1	72.2	44.3
Molise	81.5	75.0	46.5
Piemonte	78,1	72.4	45.8
Puglia	81.6	74.2	48.6
Sardegna	80.5	75.7	41.0
Sicilia	75.1	67.9	35.4
Trento	79.2	73.0	45.4
Toscana	82.4	76.7	44.2
Umbria	79.8	74.0	46.6
Valle D'Aosta	75.6	70.5	49.2
Veneto	78.2	72.7	46.5

[1] Coronavirus, le vaccinazioni in Italia regione per regione (https://lab.gedidigital.it/gedi-visual/2021/report-vaccini-anti-covid-aggiornamento-vaccinazioni-italia/).

[2] Including single dose vaccine

Table 2. SARS-CoV-2 vaccination rates (percent of population) in neighboring countries to Italy of Central Europe on January 13, 2022[1].

Country	First Dose (%)	Full (%)	Booster (%)
Slovenia	59.8	57.1	24.9
Austria	74.8	74.2	44.7
Switzerland	69.6	68.0	32.4
France	79.1	74.9	40.9
Italy	82.5	75.9	41.9

[1] Our World in Data (https://ourworldindata.org/covid-vaccinations .
